# Derivation and Validation of Predictive Factors for Clinical Deterioration after Admission in Emergency Department Patients Presenting with Abnormal Vital Signs Without Shock

**DOI:** 10.5811/westjem.2015.9.27348

**Published:** 2015-12-08

**Authors:** Daniel J. Henning, Kimie Oedorf, Danielle E. Day, Colby S. Redfield, Colin J. Huguenel, Jonathan C. Roberts, Leon D. Sanchez, Richard E. Wolfe, Nathan I. Shapiro

**Affiliations:** * University of Washington School of Medicine, Division of Emergency Medicine, Seattle, Washington; † Beth Israel Deaconess Medical Center, Department of Emergency Medicine, Boston, Massachusetts

## Abstract

**Introduction:**

Strategies to identify high-risk emergency department (ED) patients often use markedly abnormal vital signs and serum lactate levels. Risk stratifying such patients without using the presence of shock is challenging. The objective of the study is to identify independent predictors of in-hospital adverse outcomes in ED patients with abnormal vital signs or lactate levels, but who are not in shock.

**Methods:**

We performed a prospective observational study of patients with abnormal vital signs or lactate level defined as heart rate ≥130 beats/min, respiratory rate ≥24 breaths/min, shock index ≥1, systolic blood pressure <90mm/Hg, or lactate ≥4mmole/L. We excluded patients with isolated atrial tachycardia, seizure, intoxication, psychiatric agitation, or tachycardia due to pain (ie: extremity fracture). The primary outcome was deterioration, defined as development of acute renal failure (creatinine 2× baseline), non-elective intubation, vasopressor requirement, or mortality. Independent predictors of deterioration after hospitalization were determined using logistic regression.

**Results:**

Of 1,152 consecutive patients identified with abnormal vital signs or lactate level, 620 were excluded, leaving 532 for analysis. Of these, 53/532 (9.9±2.5%) deteriorated after hospital admission. Independent predictors of in-hospital deterioration were: lactate >4.0mmol/L (OR 5.1, 95% CI [2.1–12.2]), age ≥80 yrs (OR 1.9, CI [1.0–3.7]), bicarbonate <21mEq/L (OR 2.5, CI [1.3–4.9]), and initial HR≥130 (OR 3.1, CI [1.5–6.1]).

**Conclusion:**

Patients exhibiting abnormal vital signs or elevated lactate levels without shock had significant rates of deterioration after hospitalization. ED clinical data predicted patients who suffered adverse outcomes with reasonable reliability.

## INTRODUCTION

Identifying emergency department (ED) patients at risk for adverse clinical outcomes is an integral part of the early ED evaluation. Prior studies show that vital sign abnormalities, such as elevated respiratory rate, tachycardia, hypotension, and elevated shock index (heart rate [HR]/systolic blood pressure), as well as elevated lactate level identify a population of patients with a relatively higher risk of short-term adverse outcomes. 1–3 These markers are regularly assessed early in the ED evaluation to help ED providers risk stratify patients.^3^,^4^

While patients with persistent hypotension and shock are clearly at increased risk for adverse outcomes,^2^,^5^–^10^ risk stratification is more challenging in normotensive and fluid responsive patients.^2^,^8^,^11^ However, data showing the rates of adverse outcomes among those patients exhibiting markedly abnormal vital signs without shock (persistent hypotension despite resuscitation or need for vasopressors) are lacking. This risk has been assessed to a limited extent in infected patients with elevated lactate but no hypotension, who are shown to have a significant risk of adverse outcomes.^2^,^8^,^11^ Yet diagnoses are often obscured during the ED evaluation, 12 making the application of risk stratification data difficult to apply when limited to a single diagnosis. Understanding the rates, types, and predictors of serious adverse outcomes in an undifferentiated ED population exhibiting abnormal vital signs without shock would inform triage decisions, and help practitioners anticipate those patients who may require more aggressive interventions or a higher level of care at disposition.

This study evaluated undifferentiated ED patients who exhibited markedly abnormal vital signs or lactate levels, without overt shock. The objectives of this study were 1) to identify risk factors independently associated with clinical deterioration (defined as intubation, acute renal dysfunction, vasopressor use or death) occurring between hospital admission and discharge, and 2) to understand the overall risk of clinical deterioration in this population.

## METHODS

### Study Design

This was a prospective, observational cohort study of consecutive patients found to have physiologic instability in the ED. The study was conducted at a large, urban, academic ED with 55,000 annual visits. The derivation study period was November 11, 2012, to January 31, 2013. The validation cohort was from February 1, 2013, to March 20, 2013. This study was granted waiver of informed consent after expedited review from our human subjects committee.

### Participants

Inclusion criteria were all adult (age 18 or older) patients with one or more of the following vital signs abnormalities: HR>130 beats/min, respiratory rate >24 breaths/min, systolic blood pressure <90mm/Hg), shock index >1, or lactate >4mmol/L. These criteria were used based on prior studies showing the association of these vital signs and laboratory abnormalities with adverse patient outcomes.^2^,^3^,^13^–^15^ Exclusion criteria were the following: patients with tachycardia due to atrial fibrillation with rapid ventricular response or supraventricular tachycardia who were then discharged once rate control was achieved, vital sign abnormalities due to intoxication, withdrawal, psychiatric disorder, seizure, or simple trauma (ie: fracture). We also excluded patients who were discharged from the ED. Lastly, we excluded patients with shock in the ED. Shock was defined as persistent hypotension (systolic blood pressure <90mmHg) despite at least 1L of intravenous fluids or the need for vasopressors to treat hypotension.

We continuously and prospectively screened patients in the ED for possible inclusions using our information technology system. If patients had qualifying vital signs in triage, in nursing notes, or through the bedside monitors (two readings more than five minutes apart), or a serum lactate lactate >4mmol/L, then the patients were identified for possible inclusion in the study. Identified patients then underwent a confirmatory chart review to affirm the presence of inclusion criteria and absence of exclusion criteria. The confirmatory review occurred after patients were discharged and without subsequent knowledge of the hospital course.

### Data Collection

Elements of the history of present illness, triage vital signs, physical examination, past medical history, and medications were abstracted for each enrolled patient from the hospital record. Abstraction was performed by research assistants trained, directly supervised by the principal investigator, who periodically reviewed data collection for accuracy, in accordance with published guidelines. 16 The history of present illness and physical examination portions were abstracted exclusively from the emergency attending and resident charts. Basic demographics, length of stay, and disposition data were obtained from a hospital database. Likewise, all laboratory values were obtained from a hospital database.

An emergency physician adjudicated each patient’s underlying cause of instability, based on accepted definitions.^17^,^18^ Underlying causes were classified as septic, cardiogenic, hemorrhagic, hypovolemic, anaphylactic, neurogenic, and other. To determine inter-rater reliability, a second physician reviewer likewise adjudicated the first 500 charts, and the agreement between the two reviewers was found to be sufficient to proceed with a single adjudication for each patient (kappa=0.8).

### Outcome

The primary outcome was clinical deterioration occurring after hospitalization represented by the composite outcome of acute renal failure (creatinine 2× baseline), non-elective intubation, need for vasopressors, or death. This outcome could occur at any point after the patient left the ED until they were discharged from the hospital. Patients with acute renal failure or intubation in the ED qualified as having in-hospital deterioration only if they had new deterioration events after admission. Our secondary outcome was deterioration at any time after presentation to the ED, including any point during hospitalization, between ED triage and hospital discharge. The physician reviewer assessed for the presence and timing of deterioration.

We performed data analysis using SAS v9.3 statistical software (SAS Institute Inc., Cary, NC). Comparison of patient demographics and co-morbidities between patients with and without deterioration was performed with Chi-square for binary variables and Student’s T-Test for continuous variables. We constructed a multivariate logistic regression model to determine predictors of deterioration after admission was constructed. Lactate concentrations were stratified into “high” (≥4.0mmol/L), “intermediate” (4.0mmol/L> lactate ≥2.0mmol/L), and “low” (≤2.0mmol/L). We included a lactate-missing variable to impute for the 158 patients who did not have lactate measured, 19 although we do not present the results from the lactate missing variable with the model results. Other notable transformations included creating a binary variable for age ≥80 years, bicarbonate ≤20mEq/L, and a binary variable for initial HR≥130 beats per minute. We performed a univariate analysis of each covariate, using a chi-square to assess for a significant relationship. Clinical covariates with p>0.1 were removed from the modeling process. All significant variables were then used to create a logistic regression model with deterioration after admission as the outcome, and a stepwise selection process to create the final model. We validated the model using a validation cohort that consisted of the next 254 continuous patients that met study criteria, and reported the area under the curve (AUC) to assess the model on this group. The validation cohort was identified in the same manner and subjected to the same inclusion and exclusion criteria as the derivation cohort.

We used the logistic regression model as the basis for determining the sample size needed for the study. Based on the n/10 rule, we estimated that we would require at least 50 patients who suffered our primary outcome of deterioration after admission in order to include five predictors in our model. We estimated an in-hospital deterioration rate of 10% for the population based on observations from our initial data collection, and therefore determined at least 500 patients would be required to have five predictors in our model. Our validation cohort was meant to have at least half the number of patients in the derivation cohort: 250 patients.

## RESULTS

**Figure 1:**
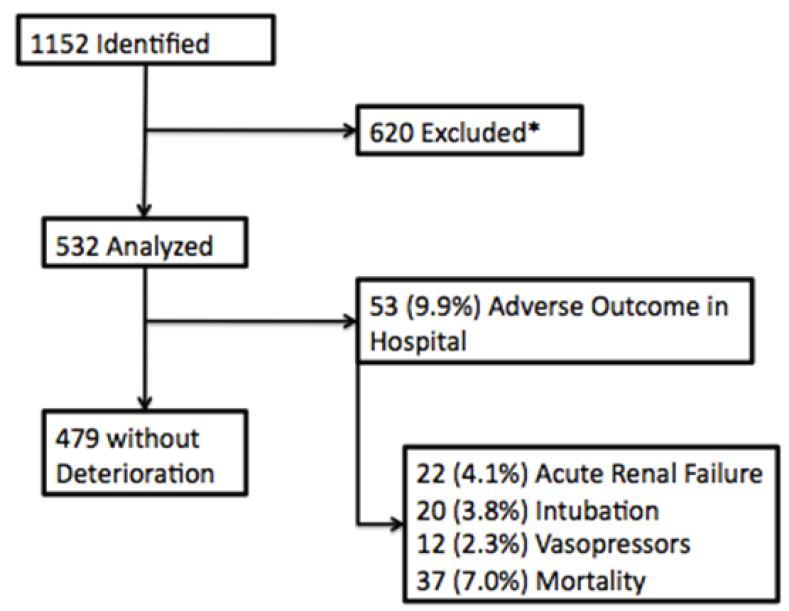
Flowchart of study enrollment, exclusions, and primary outcome. *See Supplemental Table 1 for rates of exclusion by criteria.

For our initial cohort (Figure 1), we identified 1,152 patients among 12,050 patients presenting to the ED as having vital signs meeting inclusion criteria. Of these, 620 were excluded (Supplemental Table 1), leaving 532 patients for our analysis. Of these 532 patients, we found 53/532 (9.9%) patients met the primary outcome of deterioration after admission: 22 acute renal dysfunction (4.1%, 95% confidence interval (CI) ±1.3), 20 needing mechanical ventilation (3.8%, 95% CI±1.3), 12 requiring vasopressors (2.3%, 95% CI±1.3) and 37 (7.0%, 95% CI±1.3) who died during hospitalization (Figure 1). Including those patients who had adverse outcomes in the ED, 87 (16.4%, 95% CI±3.2) patients reached the secondary deterioration outcome overall: 46 developed acute renal dysfunction (8.6%, 95% CI±2.4), 31 required mechanical ventilation (5.8% 95% CI±2.0), 12 required vasopressors (2.3%, 95% CI±1.3), and 37 died (7.0% 95% CI±1.3)( Table 1).

Table 2 shows the study demographics and displays the clinical characteristics for the study patient population. Table 3 demonstrates the clinical characteristics between the group of patients who suffered deterioration and those that did not. These tables include an unadjusted univariate measure to determine if a significant difference exists between the two groups for each covariate. The covariates that have a significant relationship (p<0.05) with the outcome are indicated. The covariates with p<0.1 from this table were included in the creation of the logistic regression model to predict in hospital deterioration.

The final logistic regression model predicting deterioration from this patient population is shown in Table 4. The covariates independently associated with adverse outcomes were the following: lactate ≥4.0mmol/L, HR≥130 beats per minute, age ≥80 years, and bicarbonate ≤20mEq/L during the ED stay. The AUC for this derivation model was 0.74, with sensitivity of 70% and specificity 63%.

**Figure 2:**
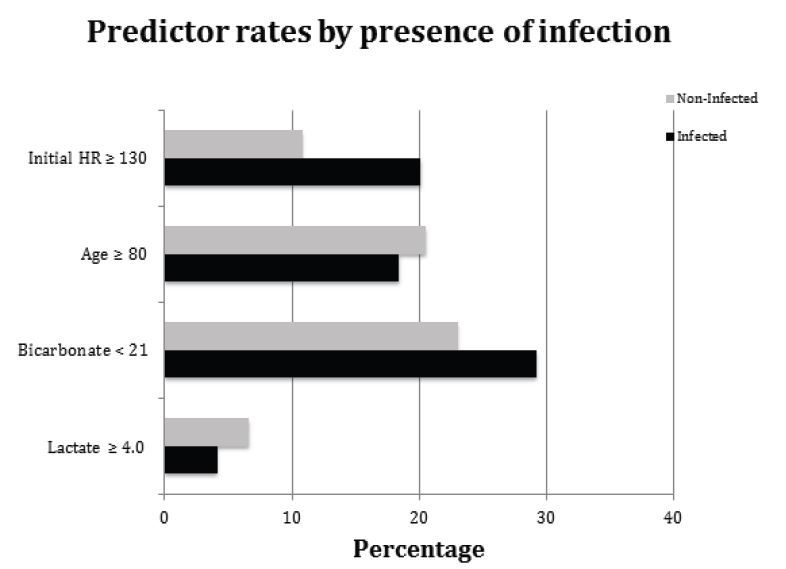
Percentage of model covariates in patients with and without infection. *HR,* heart rate

**Figure 3:**
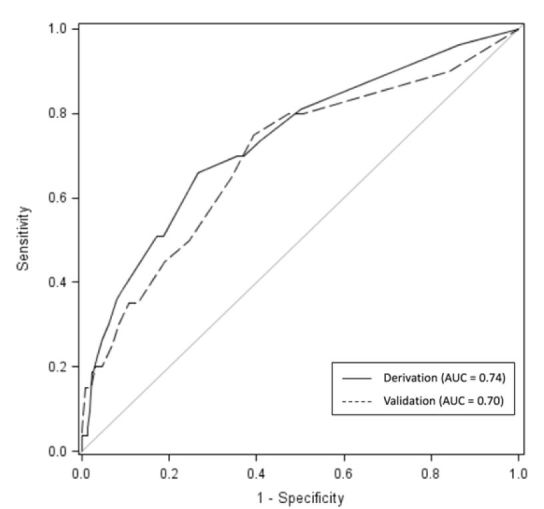
ROC curves demonstrating model performance in Derivation (solid) and Validation (dashed) cohorts. *AUC,* area under the curve

We compared the rates of covariates from the final model between infectious and non-infectious causes (Figure 2). A HR≥130 was more likely in patients with infection (p<0.01); however, no difference was found in incidence of the other model covariates between infected and non-infected groups. Then the model was re-run with infectious etiology as a binary predictor, the AUC of the model remained 0.74, and the infection term was not significant. The model was re-run on the validation cohort, with AUC=0.70. We used chi-square to test the statistical similarity of the model performed on the derivation and validation populations, which showed no difference between the model’s predictive value (p=0.70). ROC curves showing the model performance in the derivation and validation cohorts are displayed in Figure 3.

## DISCUSSION

This study of undifferentiated ED patients with markedly abnormal vital signs or elevated lactate without shock found that a significant proportion (16.4%) of these patients suffered clinical deterioration, and nearly 10% had deterioration events after being admitted from the ED. While not directly assessed, many patients in this population had improvement in hemodynamics during their ED course. This study identified independent risk factors for in-hospital deterioration so that ED and in-patient providers can better anticipate clinical course and appropriately assign level of in-patient care.

In the univariate analysis, we identified covariates associated with deterioration. Some covariates were surprisingly not associated with increased adverse outcomes, including diabetes, and congestive heart failure. Similarly, active cancer has a significant association with deterioration in the univariate analysis, but surprisingly was not significant in the modeling process. The decreased rate of deterioration in patients without any pre-existing medical problems was expected, as was the association of altered mental status with deterioration, since mental status changes can represent a form of organ dysfunction. Also, higher average lactate concentration has been associated with progression of disease,^8^,^20^ and was expected to be associated with increased event rates.

The multivariate logistic regression model allows for each independent covariate’s effect on the deterioration outcome to be determined. This model provides a few significant results for clinicians tasked with determining a disposition for ED patients. An elevated lactate is associated with increased adverse outcomes, even after controlling for patients who did not have a lactate drawn. While the prior studies of lactate concentrations have generally looked at septic patients,^2^,^7^–^9^,^11^,^20^ this result suggests that an elevated lactate level is associated with increased adverse outcomes in this general ED population. Interestingly, a lactate ≥4.0 mmol/L, occurred more frequently in the non-infected patient population, reiterating its usefulness as a predictor in undifferentiated patients,^21^,^22^ Likewise, a bicarbonate ≤20mEq/L, was a significant predictor of deterioration after controlling for elevated lactate levels, which should remind clinicians that acidosis, with or without elevated lactate levels increases the odds of adverse outcomes after hospitalization. Lastly, an initial HR≥130 beats/min increased the odds of deterioration during hospitalization. This occurred more frequently in patients with infection, which may be caused by an adrenergic response to infection or compensation for relative hypovolemia. Regardless, significant tachycardia at presentation likely reflects more serious underlying pathology compared to the other vital sign abnormalities used to triage patients in the ED.

Clinical investigations describing at-risk ED patient populations are in response to the inherent difficulty physicians face in identifying patients who will have adverse outcomes. The present study aligns with prior studies that have shown a clinically meaningful rate of disease progression, 8 progressive organ failure,^11^,^20^ and mortality^2^,^23^ in similar populations, albeit with different inclusion criteria. Prior studies have generally been limited to patients with sepsis, whereas this study includes undifferentiated patients. In prior studies that evaluated the demographics of unstable patients, the population of patients with sepsis accounts for roughly 38–43%,^24^,^25^ similar to the 46% of patients in this study with an infectious cause.

Prior studies have also attempted to identify the clinical characteristics of these at-risk populations that are associated with adverse clinical outcomes.^2^,^9^,^11^,^20^,^26^ Howell, et al., shows a strong association between lactate concentrations and mortality among patients without hypotension, although this study was limited to patients with sepsis. The degree to which this association exists in patients with infection versus those without infection remains unknown, but the present study supports the conclusions of the Howell study. In the patient population used by Song, et al., all had a lactate concentration between 2.0 and 4.0mmol/L and suspected infection. Their investigation provides other predictors of disease progression, including initial organ dysfunction and SOFA scores, but again it is limited to a population with infection.

Our model was not limited to a single underlying etiology, but included all causes of instability. This may explain the lack of significant associations between certain covariates and our outcome. Covariates more classically associated with worse outcomes in a single disease may become less significant when used to predict adverse outcomes across a spectrum of diseases. This study does not suggest that these elements would not have a significant effect on outcomes if we were evaluating a single cause of instability, and it does not replace prior studies that evaluate predictors of adverse outcomes within a single etiology.^11^,^20^ Instead, this study is meant to be applicable regardless of the underlying cause, and especially in the undifferentiated patient and early ED evaluation. By enrolling patients regardless of the underlying cause, this study bypassed the need to differentiate patients into infectious and non-infectious categories, which as stated before is inherently difficult during the initial evaluation 12.

The primary outcome of deterioration represents different types of adverse outcomes that occurred after admission to the hospital. Each type represents a meaningful clinical end-point, as well as significant morbidity and discomfort endured by the patient and increased resource utilization. Importantly, this study may assist clinicians in the early identification of patients with a higher risk of disease progression and adverse outcomes. By facilitating improved risk stratification, our findings may enable more appropriate resource allocation, thereby improving patient outcome and reducing costs associated with care.

### Limitations

This study had several limitations. We were able to collect a vast amount of clinical data, but many aspects of the history and physical examination we derived through chart abstraction possibly leading to misclassification bias. The use of a composite outcome that included four different clinical end-points may have been too inclusive. While each end-point does represent a significant adverse outcome, creating a composite outcome from very different end-points may have diminished the strength of association between different covariates and the outcome. It could be argued that death should be used as the single outcome of interest. This may become the foundation for further study; however, we believe that by including the other components of the deterioration outcome, we were able to develop a more sensitive tool that may allow practitioners to intervene earlier thereby avoiding mortality. As well, these other outcomes represent meaningful progression of disease that might be avoided with appropriate early management. Along these same lines, choosing to non-electively intubate or administer vasopressors is physician dependent to a certain extent, and there is a possibility that practice variation could affect whether or not a patient met the primary outcome.

An additional limitation was the large number of patients excluded from this study. Our screening criteria, like many triage screening tools, were overly sensitive in order to capture all moderately sick patients. Therefore, our exclusions were designed to remove those patients whose clinical stability would not be questioned by an experienced provider, i.e, patients with intoxication. Similarly, we excluded patients who were discharged from the ED. Such patients are likely to have had markedly abnormal vital signs or elevated lactate that rapidly improved with intervention suggesting a milder illness not requiring ongoing monitoring or in-patient treatment. However, this may exclude a small number of patients who would have been admitted, and therefore included in the study, if seen by an alternative provider. Also, we did not take into account how hemodynamics or lactate changed during a patient’s ED stay. While such trends clearly influence disposition decisions, the timing of repeat vital signs and lactate measurements was not able to be standardized due to our data collection methods, thereby preventing us from incorporating changes in hemodynamics and lactate into our models. Lastly, we did not assess for do not resuscitate/do not intubate (DNR/DNI) status in the ED or in the hospital. This may have had a significant effect on the rates of patients suffering adverse outcomes. However, age and other comorbidities that can be associated with a DNR/DNI order were not strong predictors of deterioration. Therefore, it is less likely that this variable represents a significant gap in the model.

## CONCLUSION

This study provides a framework for understanding the population of emergency patients who exhibit abnormal vital signs or elevated lactate in the absence of overt shock. This population is clinically relevant based on the high rate of adverse outcomes, and our analysis suggests predictors to identify those patients more likely to suffer adverse outcomes during hospitalization. Our results will help clinicians risk stratify these patients and identify the appropriate resuscitation and resource needs. A large amount of unaccounted variability remains in the model predicting deterioration, and future studies should explore the potential of newer medical devices and novel biomarkers for risk stratification.

## 

## Figures and Tables

**Table 1:** Location of deterioration events during the hospital course. Some patients had more than one deterioration event.

	Deterioration location		
	
Deterioration	ED	Inpatient	Total
Acute renal failure (%)	24 (4.5)	22 (4.1)	46 (8.6)
Intubation (%)	11 (2.1)	20 (3.8)	31 (5.8)
Vasopressors (%)	0 (0)	12 (2.3)	12 (2.3)
Death (%)	0 (0)	37 (7.0)	37 (7.0)

*ED,* emergency department

**Table 2:** Patient demographics and underlying diagnosis. Factors with p<0.1 were included as candidate variables in the multiple regression model.

	Deterioration	No deterioration	p-value
n	53	479	
Diagnosis (%)			
Sepsis	22 (47.3)	222 (46.4)	0.50
Cardiogenic	8 (15.1)	54 (11.3)	0.41
Hemorrhagic	5 (9.4)	35 (7.3)	0.58
Hypovolemic	2 (3.8)	63 (13.2)	0.05
Other	16 (30.2)	105 (21.9)	0.17
Age (95% CI)	66.8 (64.2 – 73.5)	59.8 (58.0 – 61.6)	<0.01
Female (%)	24 (45.3)	263 (54.9)	0.18
Past medical history (%)			
None	6 (11.3)	105 (21.9)	0.07
Diabetes	17 (32.1)	136 (28.4)	0.57
Coronary artery disease	8 (15.1)	78 (16.3)	0.82
Myocardial infarction	3 (5.7)	28 (5.9)	0.95
Congestive heart failure	10 (18.9)	95 (19.8)	0.87
Hypertension	24 (45.3)	210 (43.8)	0.84
Dementia	3 (5.7)	32 (6.7)	0.78
Active cancer	18 (34.0)	104 (21.7)	0.04
COPD	14 (26.4)	74 (15.5)	0.04
End stage liver disease	4 (7.6)	26 (5.4)	0.53
Chronic renal insufficiency	9 (17.0)	55 (11.5)	0.24
Dialysis	2 (3.8)	29 (6.1)	0.50
Stroke	4 (7.6)	26 (5.4)	0.53
Transplant	0 (0.0)	14 (2.9)	0.21
HIV	1 (1.9)	16 (3.3)	0.57
Anticoagulation	16 (30.2)	117 (24.4)	0.36

*COPD,* chronic obstructive pulmonary disease; *HIV,* human immunodeficiency virus

**Table 3:** Clinical characteristics of patients by outcome. Factors with p<0.1 were included as candidate variables in the multiple regression model.

History present illness (%)	Deterioration	No deterioration	p-value
Fever	15 (28.3)	159 (33.2)	0.47
Nausea/vomiting	7 (13.2)	108 (22.6)	0.12
Diarrhea	4 (7.6)	37 (7.7)	0.96
Chest pain	6 (11.3)	71 (14.8)	0.49
Shortness of breath	26 (49.1)	159 (33.2)	0.02
Abdominal pain	8 (15.1)	100 (20.9)	0.32
Cough	15 (28.3)	156 (32.6)	0.53
Dysuria	2 (3.8)	14 (2.9)	0.73
Melena	2 (3.8)	26 (5.4)	0.61
Hematemesis	3 (5.6)	5 (1.0)	0.01
Other bleeding	2 (3.7)	17 (3.6)	0.93
Rash	2 (3.7)	18 (3.8)	0.99
Physical exam (%)			
Altered mental status	6 (11.3)	42 (8.8)	0.53
Pulmonary crackles	5 (9.4)	46 (9.6)	0.97
Asymmetric lung sounds	12 (22.6)	75 (15.7)	0.19
Guaiac negative	3 (5.7)	32 (6.7	0.78
Rectal shows blood	0 (0)	9 (1.9)	0.31
Guaiac positive	1 (1.9)	9 (1.9)	0.99
Rectal shows melena	0 (0)	8 (1.7)	0.34
Abdominal distention	6 (11.3)	18 (3.8)	0.01
Abdominal tenderness	9 (17.0)	86 (18.0)	0.86
Lower extremity edema	15 (28.3)	55 (11.5)	0.01
Cellulitis	1 (1.9)	17 (3.6)	0.52
Initial vital signs (95% CI)			
Temperature (F)	98.4 (98.0–98.8)	98.7 (98.6–98.9)	0.06
Heart rate	108.8 (103.7–114.0)	106.5 (104.4–108.6)	0.38
Systolic blood pressure	110.6 (103.8–117.4)	113.8 (111.4–116.2)	0.31
Diastolic blood pressure	67.5 (62.7–72.3)	67.4 (65.9–68.9)	0.97
Respiratory rate	21.5 (20.2–22.7)	21.3 (20.7–21.8)	0.76
SaO2	97 (96.1–98.0)	97 (96.7–97.3)	0.92
Shock index	1.03 (0.97–1.1)	0.96 (0.94–0.98)	<0.01
Laboratory data (95% CI)			
Lactate	2.82 (2.43–3.21)	1.99 (1.88–2.09)	<0.01
White blood cells (count/mm^3^)	12.1 (10.7–13.6)	10.7 (10.1–11.3)	0.04
Bands (%)	6.2 (2.13–10.3)	2.54 (1.30–3.79)	0.02
Hematocrit (%)	37.5 (35.8–39.3)	36.3 (35.6–37.0)	0.16
Bicarbonate (mEq/L)	22.5 (21.0–24.0)	24.9 (24.5–25.4)	<0.01
International normalized ratio	1.68 (1.19–2.17)	1.67 (1.49–1.85)	0.96
Aspartate aminotransferase (IU/L) (mEq/dL)	97 (58.7–135.2)	58.2 (47.6–68.9)	<0.01
Alanine aminotransferase (IU/L)	57.7 (42.1–73.4)	43 (35.0–51.0)	0.09

**Table 4:** Final multivariate logistic regression predicting deterioration after hospital admission.

Predictor	β-coefficient	Std error	Odds ratio (95% CI)	p-value
Lactate ≥4.0	1.62	0.45	5.1 (2.1 – 12.2)	<0.01
Bicarbonate ≤20	0.66	0.33	1.9 (1.0 – 3.7)	0.04
Age ≥80 years	0.93	0.34	2.5 (1.3 – 4.9)	<0.01
Initial heart rate≥130	1.11	0.36	3.1 (1.5 – 6.1)	<0.01
