# Supplementary material for: Derivation and Validation of Predictive Factors for Clinical Deterioration after Admission in Emergency Department Patients Presenting with Abnormal Vital Signs Without Shock
Source: West J Emerg Med. 2015 Dec 8;16(7):1059–66. doi: 10.5811/westjem.2015.9.27348 (PMC4703194; doi:10.5811/westjem.2015.9.27348)
Supplement: Supplementary file 1 [file wjem-16-1059-s001.pdf]

Supplemental Table 1. Causes for patient exclusion

**Exclusion (N = 620)**	**n**	**%**
Age < 18	2	0.3
Psychiatric agitation	36	5.8
Indentified same patient visit twice	2	0.3
Seizure	20	3.2
Minor trauma	30	4.8
Do not meet vital signs on review	52	8.4
Atrial tachycardia discharge upon rate control	73	11.8
Intoxication	83	13.4
Withdrawal	9	1.5
Discharged	173	27.9
Shock	140	22.6
